# Accuracy of digital implant impressions obtained using intraoral scanners: a systematic review and meta-analysis of in vivo studies

**DOI:** 10.1186/s40729-023-00517-8

**Published:** 2023-12-06

**Authors:** Jie Ma, Binghua Zhang, Hao Song, Dongle Wu, Tao Song

**Affiliations:** 1 Department of Implant Dentistry, Shanghai Xuhui District Dental Center, No.500 Fenglin Road, Shanghai, 200032 China; 2 Department of Oral Maxillofacial-Head and Neck Oncology, Shanghai Ninth People’s Hospital, Shanghai Jiao Tong University School of Medicine, Shanghai, 200011 China

**Keywords:** Accuracy, Intraoral scanning, Impression, Implants

## Abstract

**Purpose:**

This systematic review aimed to investigate the accuracy of intraoral scan (IOS) impressions of implant-supported restorations in in vivo studies.

**Methods:**

A systematic electronic search and review of studies on the accuracy of IOS implant impressions were conducted to analyze the peer-reviewed literature published between 1989 and August 2023. The bias analysis was performed by two reviewers. Data on the study characteristics, accuracy outcomes, and related variables were extracted. A meta-analysis of randomized control trials was performed to investigate the impact of IOS on peri-implant crestal bone loss and the time involved in the impression procedure.

**Results:**

Ten in vivo studies were included in this systematic review for final analysis. Six studies investigated the trueness of IOS impressions, but did not reach the same conclusions. One study assessed the precision of IOS impressions for a single implant. Four clinical studies examined the accuracy of IOS implant impressions with a follow-up of 1–2 years. In full arches, IOS impression procedure needed significantly less time than conventional one (mean difference for procedure time was 8.59 min [6.78, 10.40 min], *P* < 0.001), prosthetic survival rate was 100%, and marginal bone levels of all participants could be stably maintained (mean difference in marginal bone loss at 12 months was 0.03 mm [-0.08, 0.14 mm], *P* = 0.55).

**Conclusions:**

The accuracy of IOS impressions of implant-supported restorations varied greatly depending on the scanning strategy. The trueness and precision of IOS in the partial and complete arches remain unclear and require further assessment. Based on follow-up clinical studies, IOS impressions were accurate in clinical practice. However, these results should be interpreted with caution, as some evidences are obtained from the same research group.

## Background

The passive fit of an implant-supported framework is considered a key factor in achieving long-term treatment success [1, 2]. Superstructural misfits can induce mechanical and biological complications [3, 4]. Accuracy consists of trueness and precision (International Organization for Standardisation, ISO5725-1), where trueness describes the ability of a measurement to coincide with a true or acceptable reference, and precision describes the ability of repeated measurements to coincide with the same value [5]. Steps in clinical and laboratory procedures are yet to be standardized and may influence the accuracy of the prosthesis [6]. These steps are affected by varying degrees of error, which accumulate together, resulting in a mismatch in the implant superstructure [7]. Since impression accuracy is the first step in the production of restorations, it is one of the main factors influencing decisive results [8, 9].

In recent years, digital implant impressions obtained using intraoral scanners (IOS) have been continuously developed. It relies on technologies such as triangulation, confocal lasers, and active wavefront sampling to determine the relative position of the implant [10, 11]. Compared with traditional impression technology, IOS impressions can simplify the workflow and significantly reduce time and material costs [12]. Theoretically, it may reduce the model deviation accumulated by traditional impression technology (such as impression material mixing, impression disinfection, impression storage, impression transportation, and gypsum model pouring) and can improve the accuracy and suitability of the final restoration [13–17]. The clinical indications for IOS impression are constantly increasing in patients with single tooth loss or dentition defect [18–20].

To date, there have been many in vitro laboratory investigations on the accuracy of IOS impressions [21–28]. However, in vitro studies do not completely represent in vivo condition [29]. The casts in in vitro studies had many stable reference points for scanning in the correct position. Meanwhile, many intraoral variables, such as mobile mucosa, saliva, oral humidity, and tongue movements, could affect correct digitization [30]. Therefore, this systematic review aims to evaluate the in vivo accuracy of digital implant impressions obtained using IOS.

## Methods

A systematic review was conducted in accordance with the Preferred Reporting Items for Systematic Reviews and Meta-Analyses (PRISMA) checklist. The PICO (Population, Intervention, Comparison, Outcome) question was as follows: “What are the accuracy outcomes of IOS implant impression?”.

Two independent reviewers conducted the electronic search of PubMed, EMBASE, and the Cochrane Library from 1989 to August 2023 in accordance with the PRISMA guidelines. Manual search was performed on the reference lists and conference proceedings to identify additional potential studies. The search codes are listed in Table 1.

**Table 1:** Search codes according to PICO

PICO	Codes
Population	#1 (single implant) OR (multiple/multi-unit implants)OR (partially edentulous arch/jaw) OR (complete arch/jaw) OR (full arch/jaw) OR (oral implant) OR (dental implant) OR (implant prosthesis/restorations/rehabilitation)
Intervention	#2 (digital impression) OR (intraoral scan) OR (optical impression) OR (inraoral digitizer) OR (dental scanner) OR (dental impression) OR (digital scan) OR (digital dentistry)
Comparison	#3 (conventional impression) OR (traditional impression) OR (conventional technique)
Outcome	#4 (impression accuracy) OR (trueness) OR (precision) OR (in vivo study) OR (dimensional measurement accuracy)
Search	(#1) AND (2#) AND (3#) AND (4#)

The in vivo studies investigating the accuracy (trueness, precision, or both) of IOS impressions in cases of a single implant, partial edentation, and/or full edentation were included in this analysis. In addition, only studies published in peer-reviewed journals and in English language were included in this analysis. In vitro studies, literature reviews, case reports, and technical reports were excluded. The eligibility of the selected studies was independently assessed by two reviewers and any disagreements were resolved by a third reviewer. Risk in the randomized control trials (RCTs) was assessed using the Cochrane risk of bias tool [31]. The quality of comparative studies and single-arm clinical trials was assessed using a methodological index for nonrandomized studies [32]. The following data were extracted:

- Study model (jaw; number, position, angle, depth, connection type, and impression level of implants).
- Scan (IOS type, scan body type, strategy, operator experience).
- Study design (sample size, methodological strategy to evaluate accuracy).
- Accuracy results.
- Related variables.
- Peri-implant crestal bone loss.
- Time involved in impression procedure.

The data about bone loss and time cost were combined using RevMan version5.3 (The Cochrane Collaboration, Oxford, UK).

## Results

**Fig. 1:**
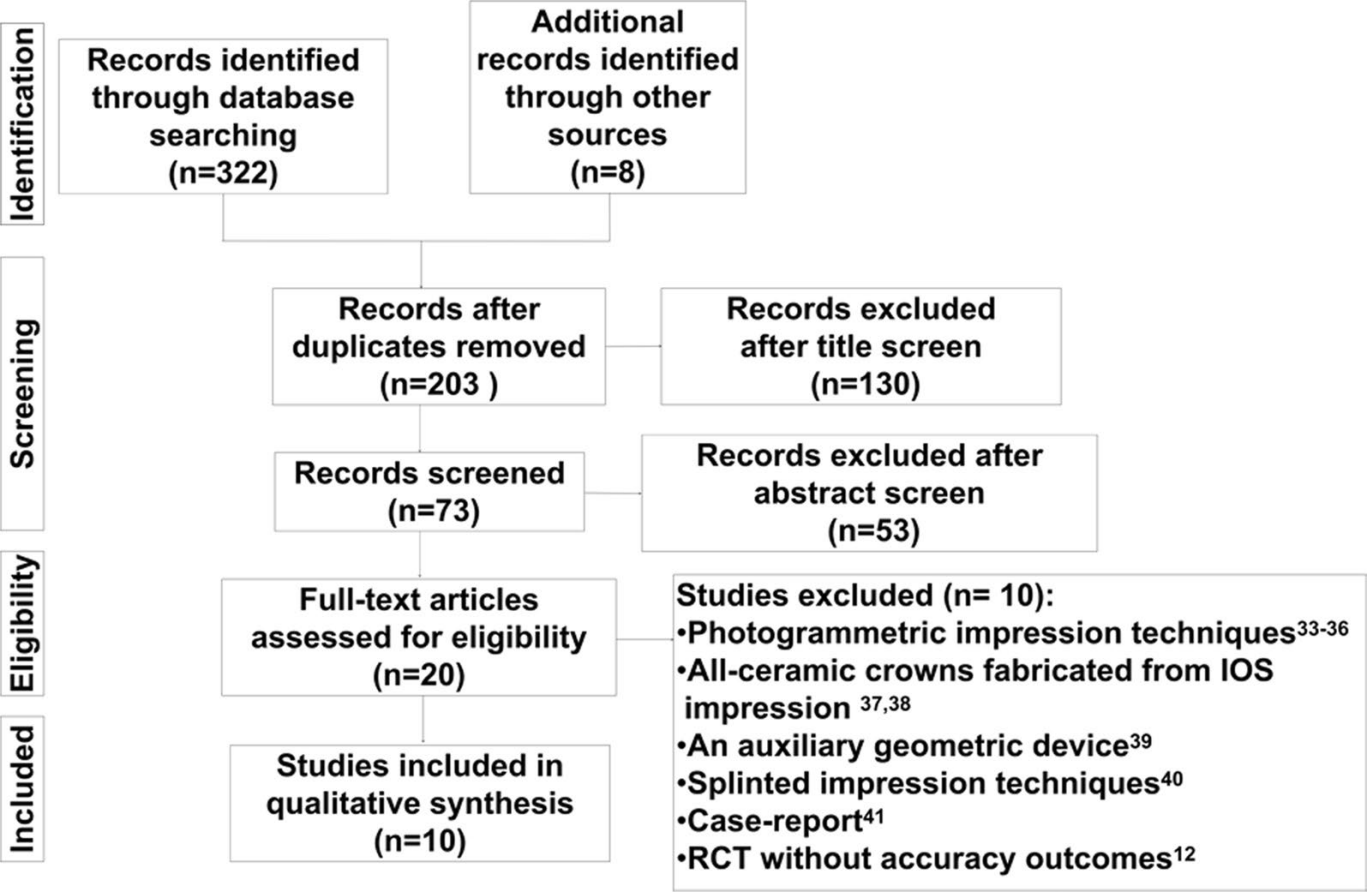
PRISMA flow diagram of search strategy

**Fig. 2:**
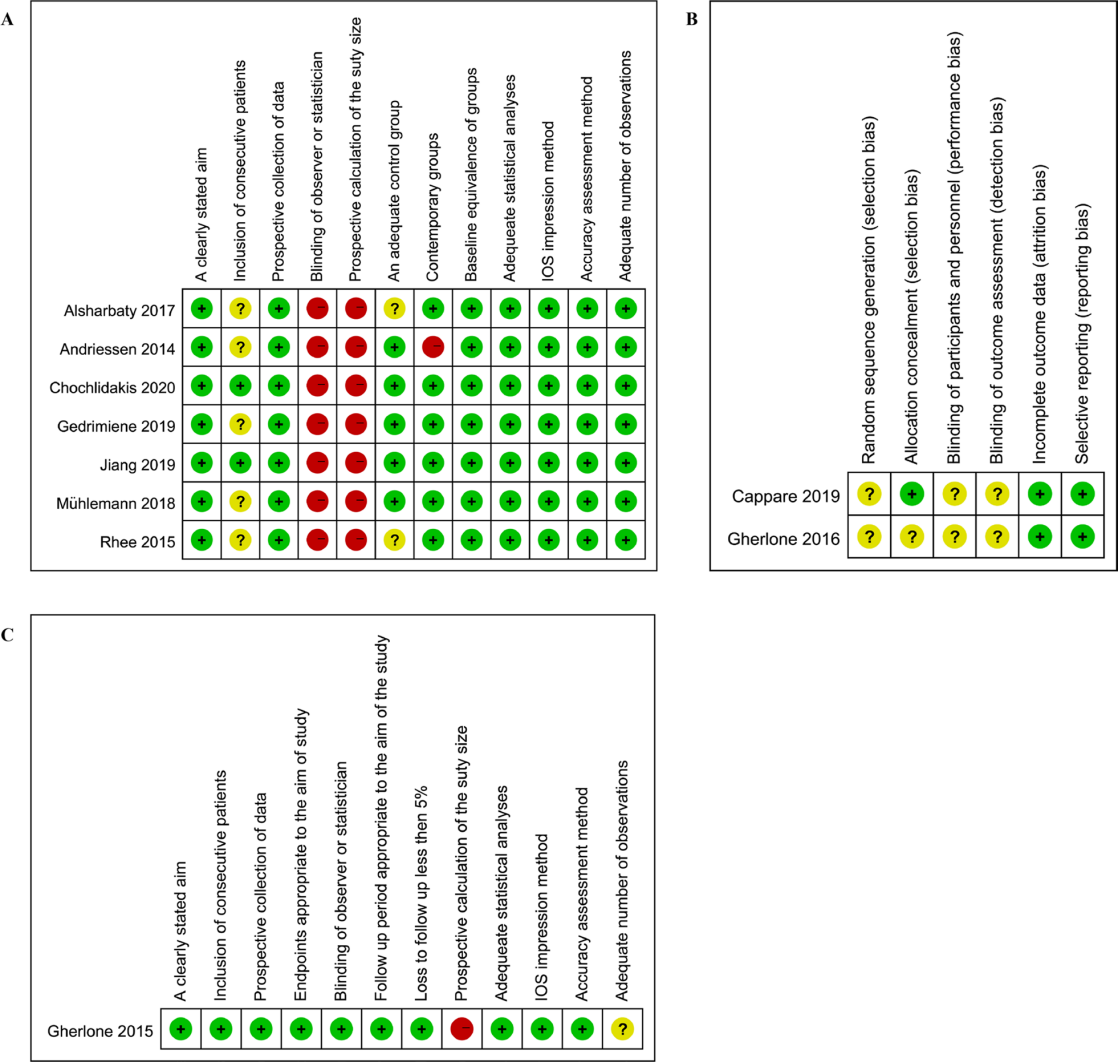
**A** The risk of bias for included comparative studies. **B** The risk of bias for included RCTs. **C** The risk of bias for included one single-arm study

In total, 322 citations were retrieved from the initial search (Fig. 1). Twenty articles were selected for full-text review. Ten studies [12, 33–41]were excluded for the reasons listed in the PRISMA flow diagram. Ten studies fulfilled the inclusion criteria and were analyzed in this systematic review [30, 42–50]. All studies included in this review were in vivo. The study characteristics are summarized in detail in Table 2. There were seven comparative studies [30, 42–47], one single-arm clinical trial [48], and two RCTs [49, 50]. The risk of bias assessment is shown in Fig. 2. All comparative studies and clinical trials clearly stated the aims, and the accuracy measurement methods were described adequately. The selection bias (random sequence generation) in the two RCTs was unclear. In all the studies, the greatest risk was associated with blinding.

**Table 2:** Characteristics of the included studies

Study (author and year)	Edentulous	Implant				Jaw
		System	No.	Position	Connection	
Rhee 2015 [42]	Single tooth loss	NA	1	36, 46	External Internal	Mandible
Mühlemann 2018 [43]	Single tooth loss	Straumann RN	1	14–17,24–27,34–37,44–47	Internal	Maxilla Mandible
Alsharbaty 2017 [44]	Partial	Dentium	2	Posterior region	Internal	NA
Gedrimiene 2019 [45]	Partial	AnyOne	2	Posterior region	NA	NA
Jiang 2019 [46]	Partial	Camlog Screw-Line	2 ~ 4	17–15,25–27,37–47	NA	Maxilla Mandible
Andriessen 2014 [30]	Complete	Straumann RN	2	NA	Internal	Mandible
Chochlidakis 2020 [47]	Complete	Straumann, BLT	4 ~ 6	NA	Internal	Maxilla
Gherlone 2015 [48]	Complete	Winsix	4	NA	NA	Maxilla Mandible
Gherlone 2016 [49]	Complete	IDI Evolution	4	NA	NA	Maxilla Mandible
Cappare 2019 [50]	Complete	CSR	6	NA	NA	Maxilla

Study (author and year)	Sample size	Impression		Operator	Scan body	IOS device
		Convention	Level			
Rhee 2015 [42]	24	Dual-arch; full arch	Implant	NA	3Shape; Raphabio	Trios mono cart
Mühlemann 2018 [43]	5	Closed-tray	Implant	One	Straumann	iTero Cadent ;Lava True Definition;Trios
Alsharbaty 2017 [44]	36	Open-tray; closed-tray	Implant	One	Dentium	Trios
Gedrimiene 2019 [45]	24	Splinted open-tray	Implant	NA	NA	Trios 3
Jiang 2019 [46]	34	Splinted open-tray	Implant	NA	Camlog	Trios
Andriessen 2014 [30]	25	NA	Implant	One	Straumann	iTero Cadent (software version 3.5.0)
Chochlidakis 2020 [47]	16	Open-tray	Abutment	NA	Straumann	True Definition
Gherlone 2015 [48]	14	NA	NA	NA	NA	Lava COS (software version 2.1)
Gherlone 2016 [49]	30	Open-tray	NA	NA	NA	Trios
Cappare 2019 [50]	50	Splinted open-tray	NA	One	CSR	CS 3600 (software version 3.1.0)

NA, not applicable

### Evaluation methods for accuracy assessment

Two main methods were used for accuracy assessment: the best-fit algorithm and absolute linear/angular deviation methods [51].

Five studies [42, 43, 45–47] tested the three-dimensional (3D) superimposition deviations between IOS and conventional impressions. Using the best-fit algorithm, they superimposed the standard tessellation language (STL) files of the IOS impression on the reference STL data to provide 3D deviations. The root-mean-square value describing the mean difference was calculated from the mean positive and negative deviations [51].

One study [30] assessed the absolute linear/angular deviation of IOS impressions. The distances and angulations between the implants were measured using IOS and conventional impression STL files, respectively. The average value of the linear/angular discrepancies was used to evaluate accuracy [51].

The evaluation method used in one study [44] was an exception. They fabricated a “true” reference model. The impression transfers were hand-tightened and splinted intraorally. They were then removed and impressed in wet gypsum. Splinted transfers in gypsum were used as the reference model. Coordinate measurement machines were used to obtain the reference data. In other in vivo studies, the implant coordinates did not fit the world coordinate system.

### Accuracy outcomes

In total, six studies [30, 42, 44–47] evaluated the trueness of IOS, and one study [43] assessed the precision of IOS.

The trueness of the IOS impression of a single implant was calculated using an in vivo study [42]. Tooth deviation was measured at some points near the implant (second premolar buccal cusp: 118.9 μm; second molar buccal cusp: 80.7 μm).

The trueness of the IOS impression in partially edentulous arches was investigated in three studies [44–46]. Among these, Alsharbaty et al. [44] (*n* = 36) found that IOS impressions produced 360 ± 46 μm 3D linear displacement, whereas pick-up impression produced only 160 ± 25 μm displacement. Significant differences were observed between the two techniques. Another study by Gedrimiene et al. [45] reported that the mean differences (*n* = 24) was 70.8 ± 59 μm which was below the possible clinical threshold of 100 μm [30]. However, they emphasized that the measured means had limited clinical relevance. Another study by Jiang et al. [46] reported opposite results. They found 3D deviation (*n* = 34) was 27.43 ± 13.47 μm, which they claimed was within the clinical acceptable range.

The trueness of IOS impressions of the full arch has been investigated in two studies [30, 47]. First, Anderiessen et al. [30] reported that a mean distance deviation was 226 μm (range: 21–638 μm) in 25 edentulous mandibles with two implants. Four of the 25 IOS impressions could not be completed because the scanned images could not be stitched together. Second, Chochlidakis et al. [47] found that the 3D deviation was 162 ± 77 μm in 16 edentulous maxillaries with 4–6 implants, and they claimed the 3D accuracy of IOS for full arch lay within the clinical acceptable threshold.

The precision of the IOS impression was assessed in one study (Mühlemann et al.) [43] for posterior single implants. They reported that the mean precision values were 57.2 ± 32.6 μm (iTero Cadent), 88.6 ± 46.0 μm (Trios 3Shape), 176.7 ± 120.4 μm (Lava True Definition), and 32.7 ± 11.6 μm (conventional impression). They concluded that conventional impressions had the greatest reproducibility of implant placement.

### Clinical studies with follow-up

**Fig. 3:**
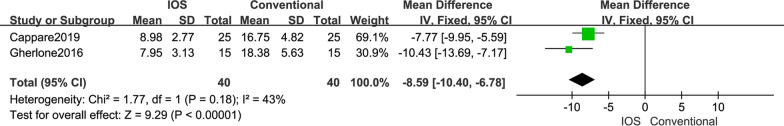
Forest plots for impression procedure time of included RCTs

**Fig. 4:**
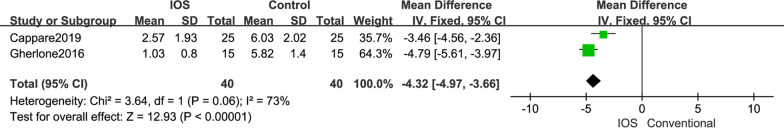
Forest plots for impression additional time of included RCTs

**Fig. 5:**
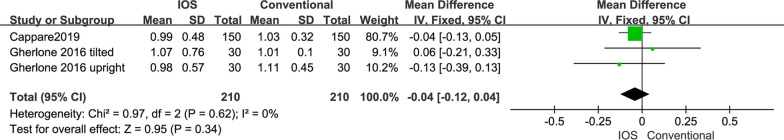
Forest plots for the marginal bone loss of included RCTs at 6 months evaluation

**Fig. 6:**
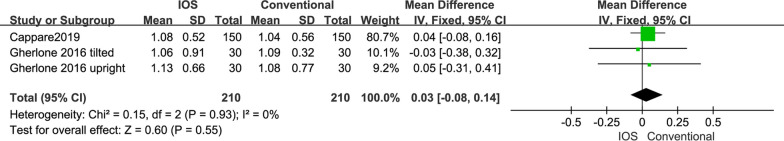
Forest plots for the marginal bone loss of included RCTs at 12 months evaluation

Four clinical studies (two prospective studies [46, 48] and two RCTs [49, 50]) assessed the accuracy of IOS impressions for implant restorations with a follow-up period of 1 to 2 years. One study (Jiang et al. [46]) reported that the time cost for IOS impression in partially edentulous patients was 17.9 ± 2.77 min. Two RCTs [49, 50] found that IOS impression for full arch spend significantly less time than conventional impression (mean difference for procedure time was 8.59 min [6.78, 10.40 min], *P* < 0.001, Fig. 3; mean difference for additional time was 4.32 min[3.66, 4.97 min], *P* < 0.001, Fig. 4). All studies reported implant and prosthetic survival rates of 100%. Three studies [48–50] for full arch found that the bar-implant connections of all definitive prostheses revealed accuracy, which were examined by intraoral digital X-ray. At the follow-up evaluation, the two RCTs [49, 50] for the full arch reported no significant difference in marginal bone loss between the IOS and conventional impression groups (mean difference at 6 months evaluation was -0.04 mm [− 0.12,0.04 mm], *P* = 0.34, Fig. 5; mean difference at 12 months evaluation was 0.03 mm [− 0.08,0.14 mm], *P* = 0.55, Fig. 6).

## Discussion

This systematic review aimed to assess the accuracy of IOS implant impressions in in vivo studies. The accuracy of the outcomes and clinical results with follow-up were analyzed in the ten included studies.

The scientific and clinical literature is scarce. In vitro equipment, such as computerized maintenance management system and laboratory scanners, cannot be used to measure actual reference data in vivo [21].

Two main methods were used for accuracy assessment: the best-fit algorithm and absolute linear/angular deviation methods. The best-fit algorithm method has been contested because it equalizes the distances of the entire surface. By comparing the two in vitro methods, Lyu et al. [51] found that the absolute linear deviation method was more efficient in detecting inaccuracies.

In the present systematic review, six studies [30, 42, 44–47] investigated the trueness of IOS. Among them, five [30, 42, 45–47] used the master model obtained from conventional impressions as an accepted reference. Master models are usually verified by passive fit evaluation techniques, such as finger pressure and the Sheffield test [52]. In addition, master models were used to fabricate definitive implant restorations. When all restorations were clinically acceptable, the master models were considered the best available references. One [44] of the six studies created a “true” reference to assess the trueness of IOS impression. However, clinically, transferring splinted copings without a common insertion path is difficult. This method of acquiring a reference model in vivo must be tested and verified in future studies.

The precision of the IOS implant impression in vivo was difficult to assess because repeated intraoral impressions were required. In the present systematic review, only one study [43] reported the precision of the three IOS devices and conventional impressions. This study resulted in 12 impressions per patient. It was necessary to extend the research period because the patients needed a break between the two impression procedures.

Currently, studies on acceptable misfit levels are not conclusive. Jemt [34, 53] assessed a screw resistance test and claimed that a limit of 150 µm would be acceptable, while some [30, 54] stated the gap at the implant–abutment interface should not be more than 100 µm. In this systematic review, diverse accuracy outcomes were found. In the partially edentulous arches, the deviation varied from 27.43 to 360 μm. These inconsistent results were probably caused by different evaluation methods, distribution of implants, IOS devices, operator experience, and scan strategies. Only two in vivo studies [30, 47] investigated the trueness of IOS impressions in patients with edentulism. They claimed opposite results. This is probably because their research designs contrasted. First, the participants in the two studies were different. The research objects of Andriessen et al. [30] were edentulous mandibles, whereas those of Chochlidakis et al. [47] were edentulous maxillae. Due to the movable tongue and unstable mucosa, there is a lack of anatomical landmarks that serve as a reference for the IOS in the mandible. In contrast, in the maxilla, the palatal mucosa is usually stable and has sufficient variable height to obtain a reference point for the IOS. Secondly, the scanning strategies used were different. Chochlidakis et al. [47] used fiducial markers in the palatal region to modify the edentulous area of the IOS, whereas Andriessen et al. [30] did not use any auxiliary geometric device. One RCT [50] in this systematic review reported satisfactory accuracy of the IOS for the complete arch rehabilitation of implants. In their study, full arches were digitally scanned with splinted scan bodies (applying orthodontic wire and composite resin). Orthodontic wire and composite resin used to splint scan bodies are auxiliary geometric devices that facilitate IOS. In addition, this RCT applied a stitching scan technique that scanned separate halves of the palate and stitched them together. Mandelli et al. [55] found that this stitching scan technique showed better accuracy than continuous scanning from one end to another. Future in vivo studies are required to assess the effects of the different IOS strategies.

Few in vivo studies have evaluated the effects of the related variables on the accuracy of IOS impression. Gedrimiene et al. [45] found that inter-implant angulation was relevant to the trueness, and Mühlemann et al. [43] found that the IOS type significantly affected the precision. The working principles of the IOSs in the present systematic review are quite different. The systems operate following the principles of confocal microscopy (Trios), parallel confocal imaging technology (iTero), active wavefront sampling technology (True Definition, Lava COS), and active-speed 3D video (CS 3600) [13]. In a systematic review, Zhang et al. compared the accuracy of different IOSs for full arch and found that Trios and CS 3600 resulted in an overall deviation below 100 μm in all of the in vitro studies, indicating reliable accuracy [21]. As the accuracy of IOS technology continues to improve, the system must gradually mature and perfect its wider application. Further in vivo studies with a new generation of IOS are required. In addition, many other related variables for the accuracy of IOS, such as inter-implant distance, implant depth, implant connection, operator experience, and scan body type, should be assessed in future in vivo studies.

Four clinical studies [46, 48–50] examined the accuracy of IOS impressions with a follow-up period of 1–2 years. Almost all of them arrived at the same conclusion: the IOS impression procedure required significantly less time than the conventional procedure, the prosthetic survival rate was 100%, and the marginal bone levels for all participants could be stably maintained. Jiang et al. [46] concluded that immediate loading of implants in partially edentulous arches with a completely digitized workflow was clinically suitable. One prospective study [48] and two RCTs [49, 50] concluded that the IOS impression for full arch implant-supported prostheses was clinically accurate. However, the two RCTs did not evaluate the distance and angular deviation of IOS impressions compared with conventional impressions using the best-fit algorithm or the absolute linear deviation method. Future RCTs should assess the deviation in the IOS and associate it with long-term clinical and follow-up observations.

The present study has some limitations. First, a small number of in vivo studies have investigated the accuracy of IOS for implant-supported restorations. Second, in the included studies, the methodological strategies to evaluate the accuracy of IOS were diverse. Third, RCTs assessing the accuracy of IOS impressions were limited, and some [48–50] of the included clinical studies were conducted by the same research group. The accuracy of IOS implant impressions must be proven by more research centers.

## Conclusions

The accuracy of the IOS impression of implant-supported restorations varies greatly depending on the scanning strategy. The trueness and precision of IOS in partial and complete arches remain unclear and require further assessment. Based on the clinical studies with follow-up, IOS impressions were accurate for clinical practice. However, these results should be interpreted with caution, as some evidences were obtained from the same research group.

## Data Availability

The data underlying this article will be shared on reasonable request to the corresponding author.

## Abbreviations

IOS: Intraoral scanning
PRISMA: Preferred Reporting Items for Systematic Reviews and Meta-Analyses
PICO: Population, Intervention, Comparison, Outcome
RCT: Randomized control trial
3D: Three-dimensional
STL: Standard tessellation language
